# A giant solitary fibrous tumor of the mesentery: a case report and literature review

**DOI:** 10.1186/s12957-014-0422-4

**Published:** 2015-02-04

**Authors:** Kiyotaka Nishida, Hideyuki Ubukata, Satoru Konishi, Jiro Shimazaki, Youko Yano, Yukio Morishita, Takafumi Tabuchi

**Affiliations:** Department of Gastroenterological Surgery, Tokyo Medical University, Ibaraki Medical Center, 3-20-1 Chuo Ami, Inashiki, Ibaraki 300-0395 Japan; Department of Diagnostic Pathology Division, Tokyo Medical University, Ibaraki Medical Center, 3-20-1 Chuo Ami, Inashiki, Ibaraki 300-0395 Japan

**Keywords:** Malignant, Mesentery, Solitary fibrous tumor

## Abstract

We report on an extremely rare case of a giant solitary fibrous tumor (SFT) of the mesentery in a 65-year-old male who was admitted to our hospital because of lower abdominal pain and abdominal fullness. Computed tomography demonstrated a well-defined solid mass of 25 × 11 cm located in the lower abdomen, which was completely resected during surgery. Histopathologically, this lesion had a heterogeneous cell population, mainly comprising spindle cells with fibrous collagen proliferation, and various other cell populations exhibiting patternless growth. Immunohistochemically, the tumor revealed strong and diffuse staining for CD34, bcl-2, and vimentin, and a high mitotic index (seven mitoses per 10 high-power fields). We diagnosed this case as an SFT of the mesentery, which is unusual according to a PubMed search that reported only nine such cases. Our case may be the largest tumor reported to date, and only one retrieved case reported recurrence, although the lesion was exceptionally large with deep invasion. Nonetheless, the lesion in our case was larger than that in the reported case of recurrence and invasive to the ileum. Since surgery, there has been no evidence of recurrence. Hence, we propose that a large SFT and high mitotic index may present risk factors for recurrence. Therefore, long-term careful follow-up is necessary in such cases, although our case exhibited few risk factors for recurrence. A follow-up at 12 months after surgery found no indications of recurrence.

## Background

A solitary fibrous tumor (SFT) is a rare mesenchymal neoplasm which often involves the thoracic cavity (pleura, lung), and less frequently soft tissues and other visceral and parenchymal organs, including the central nervous system [1-3]. Although SFTs are well described in the literature, those arising from the mesentery are very rare. Here, we report on an extremely rare case of a giant solitary fibrous tumor of the mesentery.

## Case presentation

**Figure 1:**
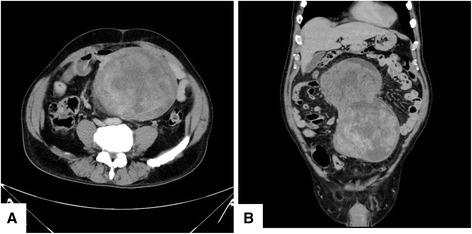
**(A) Abdominal computed tomography demonstrated a 25 × 11 cm, heterogeneous, lobulated mass in the abdominal cavity. (B)** Colonal view demonstrated lobulated mass.

**Figure 2:**
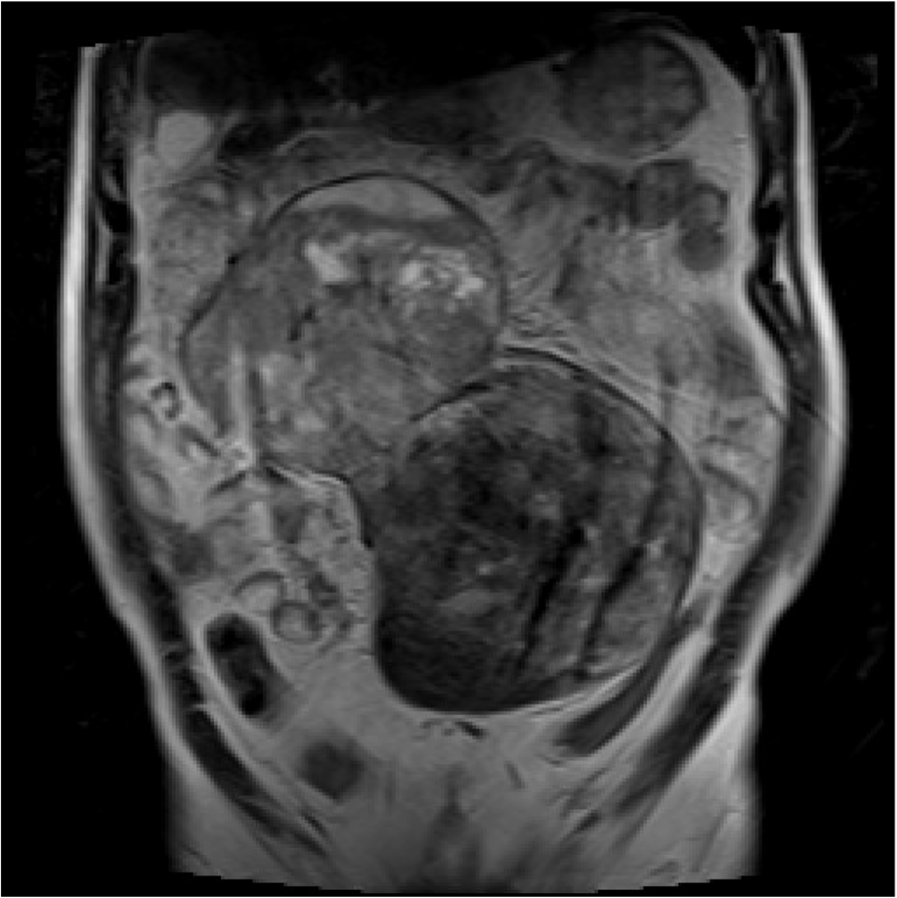
**Magnetic resonance imaging demonstrated a well-circumscribed mass with a heterogeneous high signal intensity on T2-weighted imaging.**

**Figure 3:**
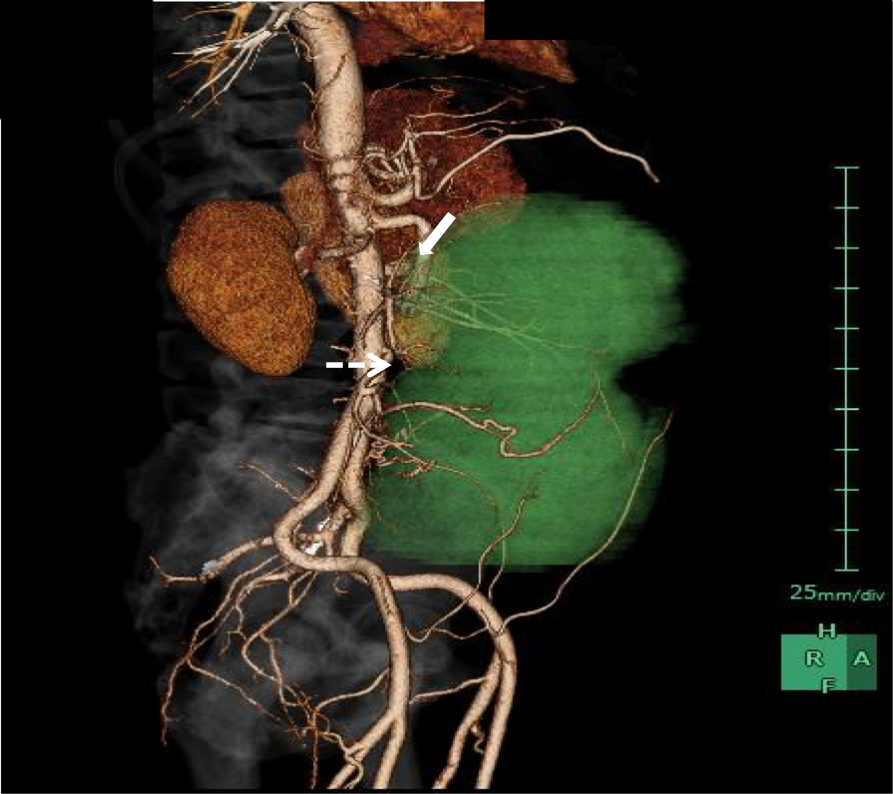
**Computed angiotomography demonstrated two feeding arteries, namely the inferior mesenteric artery (arrow) and the superior mesenteric artery (dotted arrow).** The green area demonstrates a lobulated tumor.

A 65-year-old male with no significant personal or familial medical history was admitted to our hospital 12 months after a previous visit for symptoms of abdominal fullness and lower abdominal pain. On clinical examination, routine blood results were normal, although the tumor marker cancer antigen 19-9 levels (46.4 U/mL; normal level, 37.0 U/mL) were slightly elevated, whereas those of carcinoembryonic antigen were not (2.2 ng/mL; normal level, <5.0 ng/mL). Contrast-enhanced computed tomography demonstrated a 25 × 11 cm, well-circumscribed, lobulated, heterogeneous mass in the abdominal cavity, which contained several areas of necrosis and calcification (Figure 1). Magnetic resonance imaging demonstrated a well-circumscribed mass with heterogeneous high-signal intensity on T2-weighted imaging (Figure 2). Computed angiotomography demonstrated two feeding arteries inferior to the mesentery and superior mesentery arteries (Figure 3).

We decided to perform surgery and made a midline incision of the abdomen; we resected a 25 × 13 cm firm, encapsulated mass from the mesentery of the proximal ileum. Because the tumor invaded part of the proximal ileum, we performed combined local resection of the ileum (about 30 cm) to ensure complete removal.

**Figure 4:**
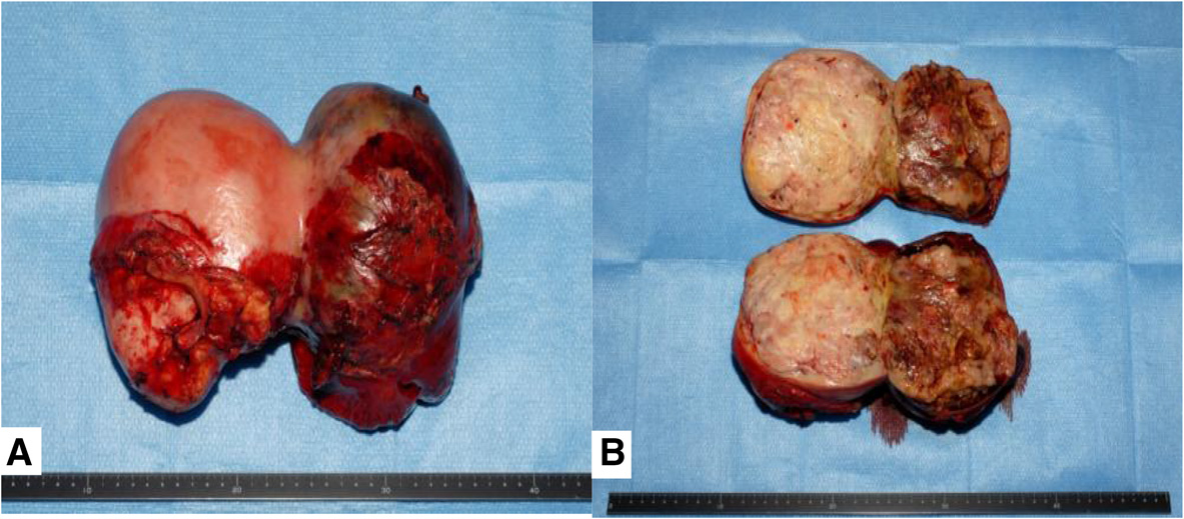
**Macroscopic examination of the tumor. (A)** The tumor (25.5 × 13 × 10 cm) was a well-defined and firm mass. **(B)** The tumor demonstrated several areas of necrotic, solid, and bleeding areas.

**Figure 5:**
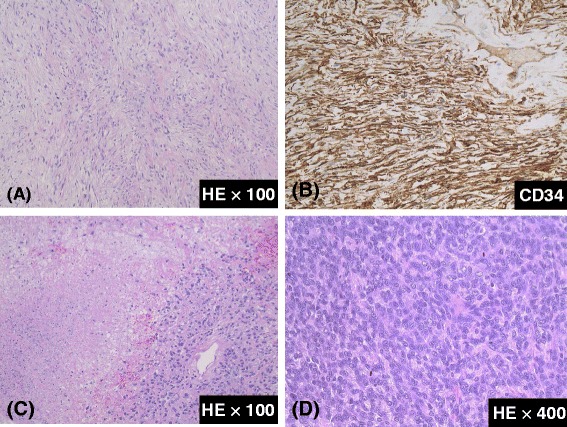
**Pathological analysis of the resected specimen. (A)** Typical areas of a solitary fibrous tumor. **(B)** CD34 immunoreactivity. **(C)** A representative necrotic area. **(D)** An image demonstrating hypercellular areas with cellular atypia and mitosis (7 mitoses/10 HPF).

Macroscopic examination of the tumor demonstrated a 25.5 × 13 × 10 cm, well-defined, firm mass with necrotic foci and a pale cut surface (Figure 4). Following histological examination, the tumor demonstrated necrosis, hypercellularity, cellular atypia, heterogeneous cell populations (comprising mostly of spindle cells that exhibit patternless growth), and fibrous collagen proliferation. Immunohistochemically, the tumor showed strong and diffuse staining for CD34, bcl-2, and vimentin; other markers, such as pan-cytokeratins, EMA, desmin, alpha-smooth muscle actin, and S-100 protein, were negative and had a high mitotic index (seven mitoses per 10 high-power fields (HPF)) (Figure 5). Based on morphological and immunohistochemical findings (a large size, hypercellular areas, cellular atypia, necrotic areas, and high mitotic index (seven mitoses per 10 HPF)), a diagnosis of solitary fibrous tumor of the mesentery with malignant potential was determined.

## Discussion

A SFT is a rare mesenchymal spindle cell neoplasm, first described by Klemperer and Rabin in 1931 [1]. Although the etiology of this lesion remains unclear, it has been recently recognized that a SFT may occur in extrapleural locations such as the lung, mediastinum, pericardium, mesentery, peritoneum, extraperitoneal spaces, nose, and paranasal sinuses [4]. However, a SFT arising from the mesentery is unusual.

A search of the PubMed database (http://www.ncbi.nlm.nih.gov/pubmed/) shows reports of only nine cases (Table 1) [5-13]. Of these reports of SFTs of the mesentery retrieved from the literature (Table 1), there was a male prevalence, with a median age of 54 years (range, 33–73 years). Although symptoms of SFTs may be unspecific, they may be related to compression or dislocation of the organs [14]. All cases were treated by surgery, as in our case. Our literature review revealed that the mesenteric SFT resected in our case may be the largest reported to date.

**Table 1:** **Solitary fibrous tumor of mesentery in English literature**

**Case no**	**Age**	**Sex**	**Complaint**	**Location**	**Tumor size (cm)**	**Treatment**	**Follow-up (months)**	**Outcome**	**Reference**
1	33	M	NP	Mesentery	NP	Surgery	NP	NP	[5]
2	68	M	Abdominal pain	S-colon mesentery	18	Surgery	NP	NP	[6]
3	53	M	Abdominal pain	Distal ileum mesentery	22	Surgery	1	Alive	[7]
4	73	M	Abdominal pain	Mesentery	25	Surgery	NP	NP	[8]
5	71	M	Painless mass	Small bowel mesentery	15.5	Surgery	12	Alive	[9]
6	41	M	Abdominal Pain	Mesentery	23	Surgery	7	Alive	[10]
7	26	M	Abdominal fullness	Proximal ileum mesentery	12	Surgery	18	Alive	[11]
8	36	M	Abdominal Pain	Rectum mesentery	15.5	Preoperative RT Surgery	NP	NP	[12]
9	59	F	Abdominal Pain	Mesentery	21	Surgery	9	Recurrence	[13]
10	65	M	Abdominal Pain	Proximal ileum mesentery	25.5	Surgery	12	Alive	Our case

NP, Not provided; M, Male; F, Female.

SFTs exhibit a wide spectrum of clinical findings; therefore, surgical resection is necessary to arrive at a final histopathological and immunohistochemical diagnosis [15]. In general, SFTs comprise various cell types, with an abundance of spindle cells exhibiting patternless growth on histopathological examination [6,15,16]. Immunohistochemically, SFTs are commonly positive for CD34, bcl-2, and vimentin, but rarely positive for S100 proteins, desmin, actin, and cytokeratins [15]. In our case, the tumor exhibited characteristics of an SFT by both histopathological and immunohistochemical analyses.

Although the majority of SFTs are histopathologically benign, up to 20% may be malignant [16]. As shown in Table 1, recurrence was reported in only one case (No. 9). In this case, the tumor was very large (21 cm) with deep invasion to the inferior vena cava and abdominal aorta, and all visible tissue was removed during surgery. In addition, part of the inferior vena cava was resected to separate it from the abdominal aorta. Therefore, although complete removal of the tumor may be achieved, recurrence remains a concern. Based on our observations, we propose that the size of a mass may be a risk factor for recurrence.

The malignant variant of a SFT generally comprises a large mass (>50 mm in diameter) and the histologic features of malignant behavior are marked by hypercellularity, necrosis, cellular atypia, and a high mitotic index (>4 mitoses per 10 HPF) [17-19]. All SFTs have the potential to become malignant; thus, gross tumor examination and counting of mitoses are recommended to assess the prognosis of any SFT [20]. In our case, histological analysis revealed characteristics of hypercellularity and invasiveness with nuclear pleomorphism and tissue necrosis. Moreover, this tumor had a high mitotic index (seven mitoses per 10 HPF). Based on these findings, we considered our case to have malignant potential.

Although the natural history of extrapleural SFTs remains unknown, long-term and careful follow-up may be necessary, especially in cases with a large tumor and a high mitotic index [21]. We followed this case for 12 months after surgery and found no indications of recurrence.

## Conclusions

Here, we reported an extremely rare case of a mesenteric SFT in which the tumor may be the largest reported to date. The tumor was completely removed during surgery; however, careful follow-up is necessary because cases with a large tumor and a high mitotic index may have malignant potential.

## Consent

Written informed consent was obtained from the patient for publication of this case report and all accompanying images. A copy of the written consent form is available for review by the Editor-in-Chief of the journal.

## Abbreviations

CT: Computed tomography
HPF: High-power fields
SFT: Solitary fibrous tumor
